# Disease association study of Autoimmune and autoinflammatory diseases by integrating multi-modal data and hierarchical ontologies

**DOI:** 10.3389/fimmu.2025.1575490

**Published:** 2025-06-04

**Authors:** Axian Liu, Yutong Su, Jinwei Zhu, Yuan-Yuan Li

**Affiliations:** ^1^ Shanghai-MOST Key Laboratory of Health and Disease Genomics, Shanghai Institute for Biomedical and Pharmaceutical Technologies, Fudan University, Shanghai, China; ^2^ Department of Rheumatology and Immunology, Ruijin Hospital, Shanghai Jiao Tong University School of Medicine, Shanghai, China

**Keywords:** autoimmune diseases, autoinflammatory diseases, diseasome, disease association, multi-modal data integration, ontology

## Abstract

**Background:**

Autoimmune and autoinflammatory diseases (AIIDs) are characterized by significant heterogeneity and comorbidities, complicating their mechanisms and classification. Disease associations studies, or diseasome, facilitate the exploration of disease mechanisms and development of novel therapeutic strategies. However, the diseasome for AIIDs is still in its infancy. To address this gap, we developed a novel framework that utilizes multi-modal data and biomedical ontologies to explore AIID associations.

**Methods:**

We curated disease terms from Mondo/DO/MeSH/ICD, and three specialized AIID knowledge bases, creating an integrated repository of 484 autoimmune diseases (ADs), 110 autoinflammatory diseases (AIDs), and 284 associated diseases. By leveraging genetic, transcriptomic (bulk and single-cell), and phenotypic data, we built multi-layered AIID association networks and an integrated network supported by cross-scale evidence. Our ontology-aware disease similarity (OADS) strategy incorporates not only multi-modal data, but also continuous biomedical ontologies.

**Results:**

Network modularity analysis identified 10 robust disease communities and their representative phenotypes and dysfunctional pathways. Focusing on 10 highly concerning AIIDs, such as Behçet’s disease and Systemic lupus erythematosus, we provide insights into the information flow from genetic susceptibilities to transcriptional dysregulation, alteration in immune microenvironment, and clinical phenotypes, and thus the mechanisms underlying comorbidity. For instance, in systemic sclerosis and psoriasis, dysregulated genes like CCL2 and CCR7 contribute to fibroblast activation and the infiltration of CD4+ T and NK cells through IL-17 signaling pathway, PPAR signaling pathway, leading to skin involvement and arthritis.

**Conclusion:**

These findings enhance our understanding of AIID pathogenesis, improving disease classification and supporting drug repurposing and targeted therapy development.

## 1 Introduction

Autoimmune and autoinflammatory diseases (AIIDs) encompass a variety of disorders characterized by the loss of immune tolerance and/or uncontrolled inflammation, resulting in either organ-specific or multi-organ damage (1). Genetic factors, environmental factors, and their interactions contribute to their etiology. It has been reported that over 10% of the population is affected by at least one of the 19 common autoimmune diseases (2). Furthermore, approximately 25% of AIID patients develop a second autoimmune disease, highlighting a notable comorbidity trend that surpasses that of other complex diseases (3).

Historically, AIIDs have been classified into autoimmune diseases (ADs) and autoinflammatory diseases (AIDs). ADs are characterized by a dysregulated adaptive immune response, while AIDs arise from innate immune imbalances and chronic inflammation (1, 4). However, recent reports have challenged this simplified classification, suggesting that AIIDs exist on a spectrum, with both adaptive and innate immune dysregulation contributing variably to different diseases (1, 5). The identification of “mixed-pattern” AIIDs characterized by co-occurrence of autoantibodies and innate markers (e.g., TLRs, inflammasomes) supports this paradigm shift in understanding AIID pathogenesis (1).

The heterogeneity and comorbidity of AIIDs complicate their mechanism understanding and classification (2, 6). Investigating disease associations across AIIDs and shared mechanisms among associated diseases could help clarify this complexity. Systematic exploration of disease associations is referred to as “diseasome” in the field of network biology, which involves the construction and analysis of disease association networks with the aim of uncovering common pathogenesis, predicting disease evolution, and optimizing therapeutic strategies (7). Early work relied on disease-related genes or pathways to establish disease associations (8, 9), which was inherently constrained by existing knowledge. The integration of Electronic Health Records (EHRs) with disease-associated genes or genetic loci helped infer genetic mechanisms underlying disease progression (10, 11). Recent advances leverage multi-omics data and clinical phenotypes to construct disease association in a data-driven manner (12–14), which enhances the ability to identify novel disease relationships and the underlying mechanisms.

However, AIID disease association network analysis remains in its nascent stage. Pioneering studies used limited data types and studied a small number of disease categories (e.g., SNPs in 6 diseases (15), comorbidity in 12 diseases (16). A recent effort explored the relationships among 26 AIIDs using a more comprehensive range of data types, including disease-related genes and gene interaction networks (17). In 2022, a disease network involving a total of 100 AIIDs was built based on their co-citation in PubMed (3). We believe that the rapid accumulation of high-quality, multi-modal data that characterize AIIDs from multiple perspectives provide an excellent opportunity to more accurately measure disease associations within AIIDs and uncover the mechanisms underlying these associations from a cross-scale perspective.

In this study, we curated disease terms from Mondo, DO, MeSH, ICD-11, and three specialized AIID knowledge bases, establishing a comprehensive AIID repository which includes 484 autoimmune diseases (ADs), 110 autoinflammatory diseases (AIDs), 14 contested diseases, and 284 diseases associated with existing AIIDs. By leveraging multi-modal data, encompassing genetic, transcriptomic (bulk and single-cell), and phenotypic data, we constructed multi-layered and integrated AIID disease association networks, which were supported by cross-scale evidence at genetic, transcriptomic, cellular, and phenotypic level. Benefiting from the novel design of ontology-aware disease similarity (OADS) strategy, our disease relationships incorporate not only multi-modal data, but also the continuous framework of hierarchical biomedical ontologies: Gene Ontology, Cell Ontology, and Human Phenotype Ontology. Network modularity analysis identified 10 robust disease communities and their key representative phenotypes and dysfunctional pathways. The single- and cross-modal disease networks around 10 highly concerning AIIDs provide deeper insights into the information flow from genetic susceptibilities to transcriptional dysregulation, alteration in immune microenvironment, and clinical phenotypes, and the mechanisms underlying comorbidity. This study could deepen our understanding of AIID pathogenesis, facilitate disease monitoring, and support the development of therapeutic strategies.

## 2 Methods

### 2.1 Disease knowledge bases and multi-modal data

We integrated seven disease databases: Mondo (Monarch Disease Ontology, v2024-04-02, 26,961 diseases, https://monarchinitiative.org), DO (Disease Ontology, v2024-03-26, 15,777 diseases, https://disease-ontology.org), MeSH (Medical Subject Headings, v2023-11-16), ICD-11 (International Classification of Diseases 11^th^, 2023), and three specialized AIID databases: AA (the Autoimmune Association, 151 diseases, https://autoimmune.org), ARI (Autoimmune Registry, Inc., 167 diseases, https://www.autoimmuneregistry.org), GAI (the Global Autoimmune Institute, 160 diseases, https://www.autoimmuneinstitute.org).

Gene expression data were curated from Affymetrix U133A platforms (GPL570/96/571) in GEO, filtered by disease/control groups ≥5 samples each, and tissue sources (PBMCs/whole blood/skin). scRNA-seq data were obtained from five major platforms (GPL24676/18573/16791/11154/20301) through GEO searches with disease synonyms.

### 2.2 Calculation of the normalized AIID classification score

To capture both the direction and confidence of each classification source, we extended the original +1/–1 AIID Classification Score (ACS) into a continuous, weighted metric on [–1, +1]. For each disease and each source *i*, let *s_i_* denote classification as autoimmune, unclassified or autoinflammatory, and let *w_i_* be the source’s confidence weight (based on coverage, update‐frequency and community endorsement; Mondo = 1.0, DO = 0.8, MeSH = 0.7, ICD = 0.7, expert panel lists = 1.0, AA/ARI/GAI = 1.0). We then compute:


(1) $$ACS_{norm}=\frac{\sum_{i}w_{i}S_{i}}{\sum_{i}w_{i}}$$

and report these values (**Supplementary Table 1**) alongside the original binary ACS.

### 2.3 Calculation of ontology-aware disease similarity

Differential co-expression analysis used DCGL package (18) with Z-score normalized dC values, retaining top20 Gene Ontology terms per disease.

Disease genes, including genetically associated disease genes obtained from OMIM and dysregulated genes (DCGs), calculated by DCGL, were mapped to GO (Biological Process). DCGs were weighted by normalized dC values, retaining top20 GO terms per disease.

scRNA-seq data were processed through Seurat (QC/normalization/clustering) with SingleR-based cell annotation (19).

Phenotypic terms were extracted from Human Phenotype Ontology (HPO).

Ontology similarities were calculated using Wang method for GO/HPO (20), CellSim for Cell Ontology (21). Disease similarity was computed via FunSimAvg (22), averaging bidirectional GO term assignments.

### 2.4 Calculation of drug-based disease similarity

Drug-disease relationships from five databases (DrugBank/DrugCentral/TTD/PharmGKB/CTD) were filtered to retain SMILES-defined drugs (23). Structural similarity between drugs was computed using RDKit, and drug-based disease similarity was derived via FunSimAvg aggregation.

### 2.5 Evaluation of disease similarity by permutation

Disease-term mappings were shuffled 500× while preserving term counts/distributions. Null distributions were generated by recalculating similarities.

### 2.6 Disease network construction and modularity analysis

Python/NetworkX was used to build disease networks with edge’s similarity scores > 90th percentile and p < 0.05. Disease modules/communities were detected by Hierarchical clustering (Ward’s method) (24) and Leiden algorithm (resolution =1.0) (25).

### 2.7 Topological analysis of disease networks

NetworkX library was used to calculate centrality measures (degree, betweenness, closeness, eigenvector centrality), clustering coefficient, transitivity, k-core, network diameter and shortest path lengths.

### 2.8 Evaluation of power-law characteristics

The network degree distribution was fitted to a power-law model using powerlaw library (26) to evaluate if a network display power-law behavior.

### 2.9 WiND

The within-network distance (WiND) (27), the mean shortest path length (d(i,j)) among all links in the network (k), was defined to describe the closeness of a network:


(2) $$WiND=\frac{\sum_{i,j\in c}d(i,j)}{k}$$

### 2.10 Identification of significant features in disease community

To determine the representative features of diseases within communities, we counted the frequency of each feature in a given cluster relative to all other clusters. For each feature, a 2×2 contingency table was constructed, and Fisher’s exact test was used to assess its enrichment, using p-values < 0.05 and Benjamini-Hochberg adjusted p values (28).

### 2.11 Similarity network fusion

Disease similarity matrices from different modalities were integrated using the snf.snf function from SNFpy Package. The key parameters were set as K=10 (number of neighbors for information propagation) and t=20 (number of iterative fusion steps) (29).

### 2.12 Minimum spanning tree construction

The similarity matrix and corresponding significance matrix were used to filter out non-significant similarity values (top 10% & p<0.05). A distance matrix was then calculated from the filtered similarity values as Distance = 1-Similarity. Using this distance matrix, a Minimum Spanning Tree (MST) was constructed to capture the most parsimonious connections among diseases based on Prim’s algorithm (30).

## 3 Results

### 3.1 Construction of an integrated repository of autoimmune and autoinflammatory diseases and their associated diseases

Considering the discrepancies in Autoimmune and autoinflammatory diseases (AIIDs) across various disease classification systems, we collected AIID terms from four mainstream resources including Mondo, DO, MeSH, ICD-11, and three specialized autoimmune knowledge bases, including AA, ARI, AGI (see Methods for the details of all databases). This allowed us to construct a repository that comprehensively covers a wide range of AIIDs.

The initial repository of AIIDs was derived from Mondo, the most extensive and coherent ontological representation of human diseases. In this repository, autoimmune diseases (ADs) were retrieved using the Mondo ID MONDO:0007179 and its subclasses, encompassing 255 diseases. Similarly, autoinflammatory diseases (AIDs) were retrieved using the Mondo ID MONDO:0019751 and its subclasses, comprising 83 diseases. Some diseases were classified as both ADs and AIDs in the original databases; for example, Blau syndrome (MONDO:0008523) is included in both categories.

Using Monde-derived initial repository, including ADs and AIDs, as a reference framework, we incorporated additional AIIDs from DO, MeSH, and ICD-11. Among them, DO contains 166 ADs. Although all of these are included in Mondo, 80 of them are not classified as AIIDs in Mondo. Therefore, these 80 diseases were added to the AD category of the initial repository. Since DO does not have a specific category for AIDs, we extracted 14 AIDs using keyword matching with the terms “autoinflammation” or “autoinflammatory”, such as familial cold autoinflammatory syndrome and proteasome-associated autoinflammatory syndrome. All of these diseases are classified as AIDs in Mondo, so they do not alter the composition of the initial repository. Similarly, MeSH includes 73 ADs and 9 AIDs, of which 19 ADs were added to our AIID repository. Unlike the previous cases, ICD-11 includes classifications for both ADs and AIDs, with 109 and 10 members, respectively, which contributed additional 41 ADs and one AID to our AIID repository.

Next, we incorporated three specialized autoimmune knowledge bases: AA (151 diseases), ARI (167 diseases), and GAI (160 diseases), which collectively contain 208 non-redundant ADs. Among them, 92 disease entries not yet included in our AIID repository were added.

Moreover, clinical experts proposed a curated repository of 62 AIDs based on their clinical experience; of these, 38 were not yet included and subsequently added to our AIID repository. Ultimately, we constructed a comprehensive repository of 608 AIID terms.

In addition to discrepancies across classification systems in defining AIIDs, inconsistencies in the differentiation between ADs and AIDs were also observed. Adult-onset Still’s disease exemplified this divergence (AID in Mondo vs. AD in MeSH/DO), while Blau syndrome demonstrated classification ambiguity within Mondo (classified as both AD and AID in Mondo).

The process of constructing the AIID disease repository clearly demonstrates that there are substantial discrepancies across resources in defining AIIDs and differentiating AIDs from ADs. To quantitatively evaluate the discrepancies across systems, we propose the AIID Classification Score (ACS) to measure classification agreement across these systems. First, diseases categorized as both ADs and AIDs, either within the same system or across different systems, are designated as Contested Autoimmune and Autoinflammatory Diseases (Contested AIIDs, CAs). Among the 608 AIID terms, 14 were categorized as CAs. Then, each disease was assigned an ACS score based on its categorization in different systems. Specifically, being classified as AD by one classification system earns +1 point, while being classified as AID earns -1 point. Among diseases excluding CAs, those with ACS ≥1 were recorded as ADs, while those with ACS ≤-1 as AIDs.

**Figure 1:**
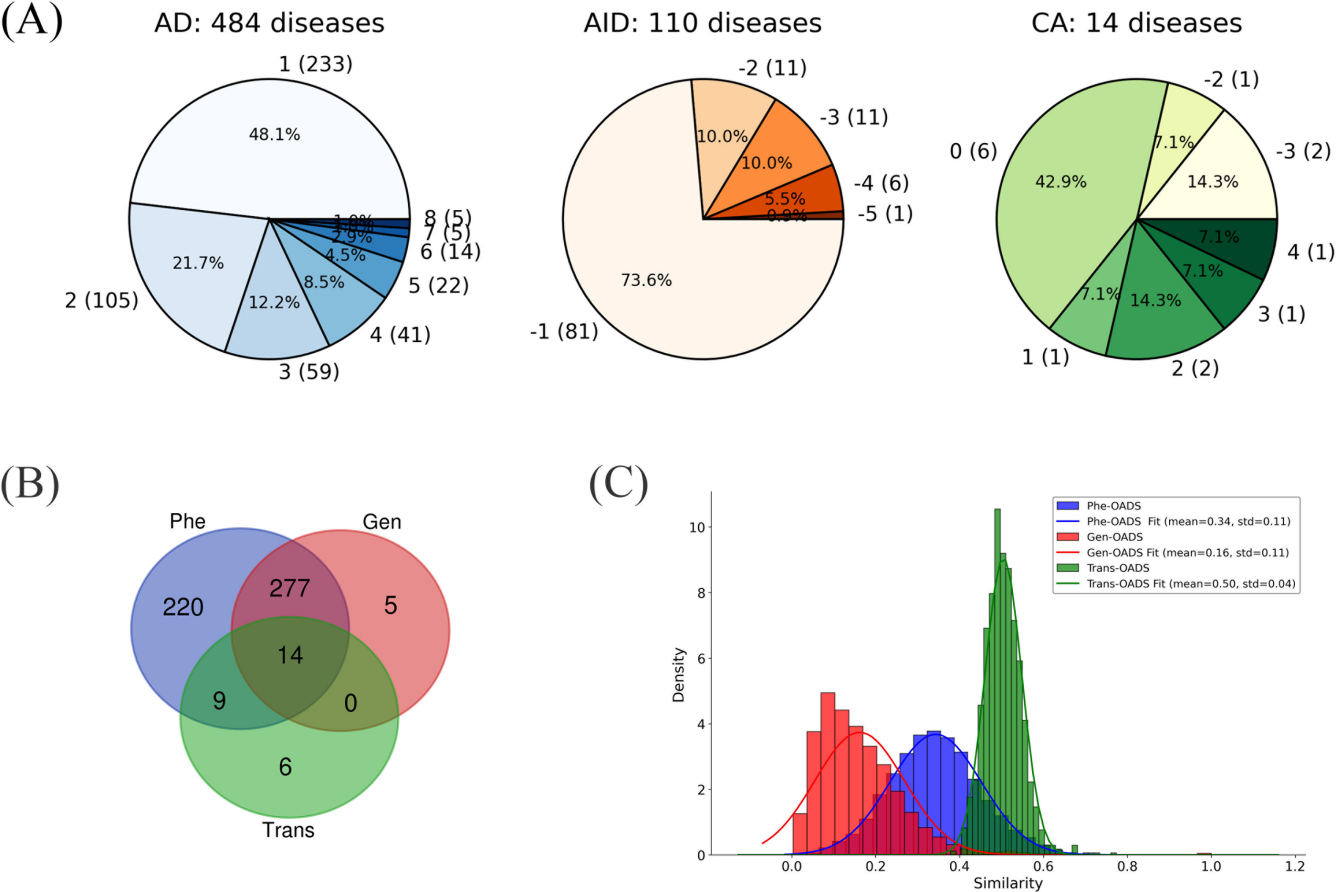
**(A)** Category Distribution of AIIDs (ADs, AIDs, CAs) with ACS Scores and Disease Counts (Disease counts in parentheses; ACS scores outside parentheses); **(B)** Overlap of diseases with different modality data; **(C)** Distribution of each Ontology-Aware Disease Similarity.

As shown in **Figure 1A**, 48.1% of 484 ADs and 73.6% of 110 AIDs received a score of 1 or -1. This means that over half of these diseases (314 out of 594) were recognized as AIIDs (either AD or AID) by only one classification system, suggesting that a consensus has yet to be reached. Complementing our binary ACS tally, we computed a normalized AIID Classification Score (ACS_norm_; see Methods and Equation 1) that integrates source‐specific weights into a continuous [–1,+1] scale, where values near +1 (–1) indicate strong autoimmune (autoinflammatory) bias. ACS_norm_ values for all diseases are provided in **Supplementary Table 1**. This assessment provides an overview of the AIID collection across multiple classification systems, serving as a foundation for building consensus and resolving discrepancies.

Given the significant discrepancies in the inclusion of AIIDs across different classification systems, we further expanded our AIID repository by incorporating diseases with autoimmune-related phenotypes that are not explicitly classified as AIIDs in existing resources. These diseases, termed AIID-associated diseases (AAs), were identified based on the “autoimmunity” phenotype (HPO ID: HP:0002960) in the Human Phenotype Ontology (HPO) database. In total, 416 diseases with the “autoimmunity” phenotype were retrieved from HPO; of these, 132 were already included in the previously compiled repository of 608 AIIDs. The remaining 284 diseases, categorized as AAs, were added to supplement the AIID repository, further enhancing its comprehensiveness and coverage.

In total, we constructed an integrated repository of AIIDs and their associated diseases (AAs), which includes 484 ADs (54.3%), 110 AIDs (12.3%), 14 contested AIIDs (CAs; 1.6%), and 284 associated AIIDs (AAs; 31.8%). This disease repository serves as the basis for subsequent disease network construction and analysis (**Supplementary Table 1**).

### 3.2 Calculation of ontology-aware disease similarity

In order to quantitatively estimate pairwise disease associations within our AIID list, we first collected various types of disease-related data, including genetically associated disease genes, transcriptomic data, and phenotypic data, which enable a more comprehensive and in-depth characterization of diseases. Unlike existing data-driven strategies for measuring disease similarity, we designed an Ontology-Aware Disease Similarity (OADS) strategy to integrate the hierarchical biomedical ontologies with the collected multi-modal data.

Specifically, at the genetic level, genetically associated disease genes were mapped to GO. Then, GO term similarity was calculated (20), and pairwise disease similarities were computed by integrating the GO terms assigned to each disease (22). Instead of merely counting the number of overlapping genes or pathways in previous disease association studies (31), our genetic-level ontology-aware disease similarity (Gen-OADS) strategy measures the association between diseases based on the functions of genetically associated disease genes and the hierarchical relationships among those functions. At the transcriptomic level, differentially co-expressed genes, or dysregulated genes, for each disease were mapped to GO. Similarly, GO term similarity and disease similarity were calculated, generating transcriptional-level ontology-aware disease similarity (Trans-OADS). Differently from Gen-OADS, GO term similarity was further weighted by the average differential normalized co-expression (dC) value of their associated genes before it was used to estimate disease similarity. At the phenotypic level, disease phenotypes were extracted from HPO to compute semantic similarity between HPO terms and then phenotypic-level ontology-aware disease similarity (Phe-OADS). In this manner, we incorporated the hierarchical biomedical ontologies, GO and HPO, to multi-modal information that span genetic susceptibility, transcriptional dysregulation, and clinical manifestation, achieving the fusion of multi-modal data and continuous multi-level knowledge. Due to data availability constraints, we eventually obtained semantic similarity matrices for 296 diseases supported by genetic evidence (Gen-OADS), 57 diseases supported by transcriptional dysregulation (Trans-OADS), and 520 diseases supported by clinical phenotypes (Phe-OADS) (**Supplementary Table 2**), with the overlap of disease types across modalities illustrated in **Figure 1B**.

Furthermore, by comparing the similarity distributions across modalities, we found that the average similarity exhibits the following trend: Trans-OADS > Phe-OADS > Gen-OADS. Notably, associations based on transcriptional dysregulation are more concentrated (**Figure 1C**), suggesting that the Trans-OADS measure is more sensitive in capturing disease similarity, which is likely due to DCGL’s ability to detect dysfunctional regulation (32, 33). In contrast, the Gen-OADS distribution has a lower mean and is skewed toward zero, probably attributed to the uneven distribution of genetic association knowledge across diseases.

### 3.3 Construction of single-modal disease networks and evaluation of their consistency with traditional classification

**Figure 2:**
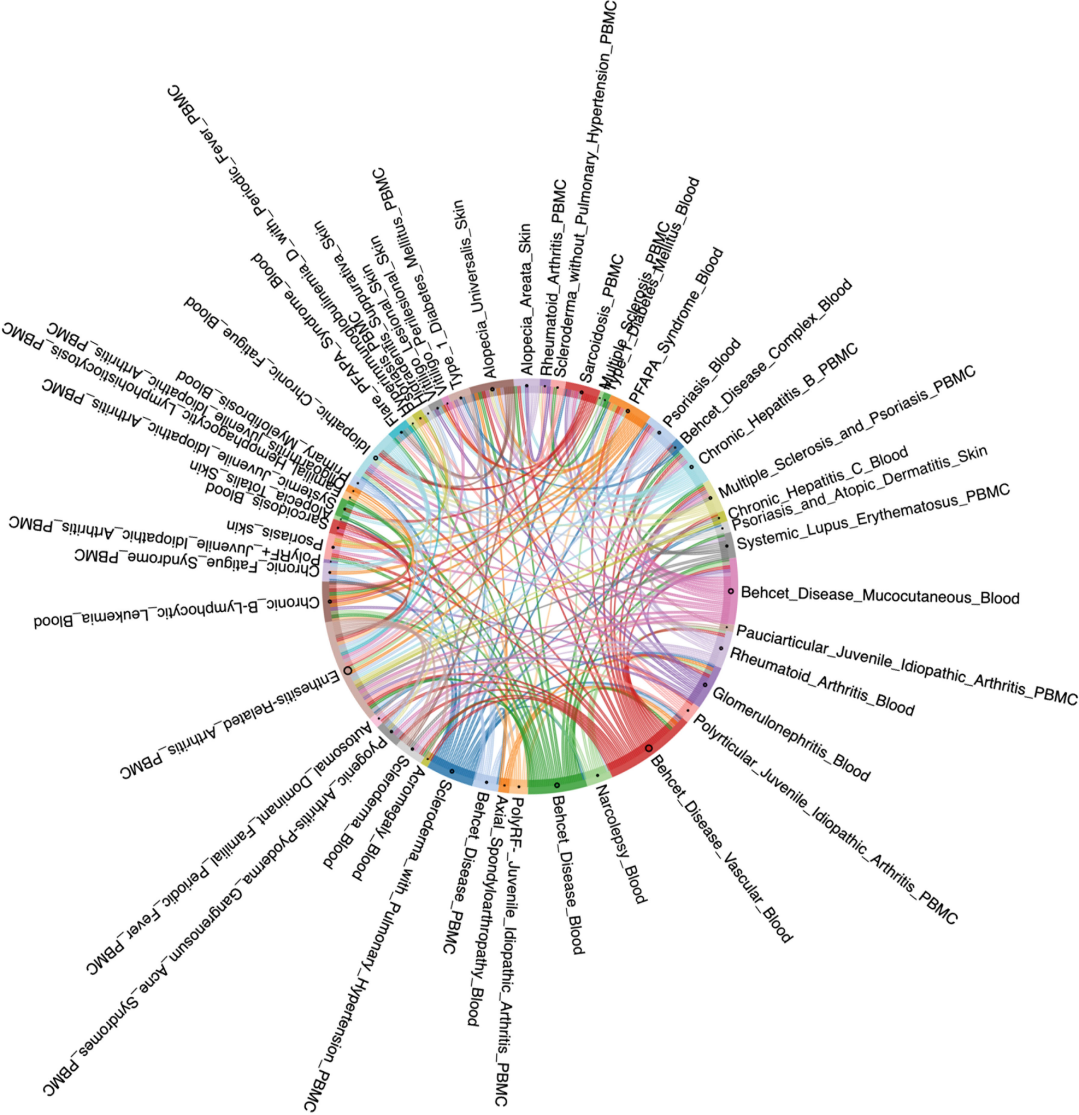
Transcriptomic disease network (Trans-DN) supported by dysregulation evidence.

Based on the three levels of OADS, we constructed disease association networks for AIID at the genetic, transcriptomic, and phenotypic levels by identifying statistically significant disease associations with similarity values above the 90th percentile and permutation p-values below 0.05. This resulted in three single-modal AIID networks: the Genetic Disease Network (Gen-DN) supported by genetic evidence, the Transcriptomic Disease Network (Trans-DN) supported by evidence of dysregulation (**Figure 2**), and the Phenotypic Disease Network (Phe-DN) supported by clinical symptom data. Topological analyses (**Table 1**) show that both Gen-DN and Phe-DN exhibit scale-free, small-world properties with power-law degree distribution (34) (**Supplementary Figures 1A, B**), whereas Trans-DN exhibits characteristics of a random network (**Supplementary Figure 1C**). Given the smaller number of diseases represented in the transcriptomic data, the true distribution characteristics of this network layer remain to be fully elucidated once more data become available.

**Table 1:** Topological properties of Phe-DN, Gen-DN, and Trans-DN.

Properties	Gen-DN	Trans-DN	Phe-DN
Number of Nodes	293	45	514
Number of Edges	3300	92	12179
Average Degree	22.526	4.089	47.390
Network Density	0.077	0.093	0.092
Transitivity	0.370	0.297	0.486
K-core Size	99	11	73
Average Clustering Coefficient	0.467	0.324	0.536
Average Shortest Path Length	2.420	2.766	2.375
**Degree distribution**	**Scale-free network**	**Random network**	**Scale-free network**

Bolded text “Degree distribution” indicates key topological metrics, with corresponding visual representations shown in **Supplementary Figures 1A-C**.

Notably, Phe-DN has more nodes and edges, as well as higher average degree, density, clustering coefficient, and transitivity, indicating extensive phenotypic associations among AIIDs at the phenotypic level. In contrast, although Gen-DN shows lower average degree and clustering than Phe-DN, it has the largest K-core, suggesting a large and stable core of tightly interconnected genetic associations among diseases.

It is noteworthy that SLE is among the top 10 nodes with the highest degrees across all three networks (**Supplementary Table 3**), consistent with reports of its significant comorbidity relationships (35, 36). Our three single-modal networks, along with the detailed supporting evidence, provide a foundation for further hypotheses on the mechanisms underlying these associations.

To investigate the consistency between our AIID networks and traditional disease classification knowledge, we examined the connectivity of diseases grouped by four Mondo categories - “Autoimmune Diseases (Mondo-AD)”, “Immune Deficiency Diseases (Mondo-IDD)”, “Rheumatic Diseases (Mondo-RD)” and “Autoinflammatory Diseases (Mondo-AID)”. WiND values of the subnetworks corresponding to certain categories (Category WiND) and that of the whole network (Network WiND) were calculated, from which WiND ratios were derived (see Equation 2). As shown in **Table 2**, the WiND ratios for all categories in the Gen-DN, Trans-DN, and Phe-DN were below 1, except for AID in Gen-DN, suggesting that AIID subcategories tend to form clusters in our networks. The slightly elevated ratio for AID in Gen-DN, similar to the lower mean similarity in Gen-OADS, likely reflects uneven accumulation of knowledge on genetic associations.

**Table 2:** The bolded WiND value greater than 1 represents a unique case originating from the AID-WiND subnetwork.

Network	Mondo Category	Disease Count	Category WiND	Network WiND	WiND Ratio	Significant Pair Count
Gen-DN	AD	30	2.262	2.420	0.935	160
	AID	**54**	**3.069**		**1.268**	**262**
	RD	11	1.756		0.725	34
	IDD	62	2.378		0.983	466
Trans-DN	AD	8	1.000	2.766	0.362	12
	AID	9	1.545		0.559	24
	RD	7	1.250		0.452	4
	IDD	2	–		–	*
Phe-DN	AD	93	2.243	2.375	0.944	1208
	AID	61	1.847		0.778	1222
	RD	26	1.674		0.705	266
	IDD	72	1.450		0.611	2872

*indicates that the two diseases are not significantly associated.

To explore the consistency and complementarity of our three single-modal AIID networks, or the three OADS measures in disease association representation, we selected 91 disease pairs among 14 diseases that contain all three data modalities. Taking the similarity ranking of disease pairs in the Phe-OADS modality (from rank 1 to rank 91) as a reference, we calculated Kendall’s Tau coefficients (37) for the disease pair rankings across the three modalities to assess their consistency. Our analysis revealed low correlations (-0.001 for Phe-OADS vs. Gen-OADS, 0.110 for Phe-OADS vs. Trans-OADS, and 0.154 for Trans-OADS vs. Gen-OADS; **Supplementary Figure 1D**), highlighting the inherent differences between modalities and emphasizing the necessity of a multi-modal approach in disease association studies.

### 3.4 Network modularity analysis

To analyze the network structure and uncover intrinsic connections between diseases, we employed two complementary clustering methods: hierarchical clustering (HC) and the Leiden algorithm (25). Specifically, HC provided global dendrograms, while Leiden pinpointed robust disease communities.

Clustering analyses were conducted on Gen-DN, Trans-DN, and Phe-DN using HC and Leiden, respectively. This process resulted in 6 to 17 clusters for each network. We further assessed the alignment of these clusters with the four disease categories concluded above, AD, AID, CA, and AA (**Supplementary Figure 1E**). The results indicate that most clusters are heterogeneous, which contain diseases from multiple categories with only five clusters consisting entirely of AD diseases and one cluster containing a single AID disease. See **Supplementary Table 3** for detailed clustering results and network characteristics.

Recognizing that robust disease modules - those insensitive to data modalities and clustering methods - likely reflect true disease connections, we evaluated clustering consistency using the adjusted Rand index (ARI). Since only 14 diseases overlapped among the three single-modal networks, we compromised to focus on Gen-DN and Phe-DN, which share 291 diseases. After excluding 9 diseases that did not meet our criteria, 282 diseases were analyzed. The ARI between Phe_Leiden and Phe_HC was 0.32, and between Gen_Leiden and Gen_HC it was 0.22, indicating moderate consistency between clustering methods; however, the cross-modal ARIs (0.05 for Phe_HC vs. Gen_HC and 0.04 for Phe_Leiden vs. Gen_Leiden) were very low (**Supplementary Figures 2A, B**). These findings highlight the complementarity of multi-modal data in capturing disease characteristics again. Furthermore, by identifying diseases that consistently clustered across the four combinations, we delineated 10 robust disease communities (each containing at least four diseases).

Among the identified communities, Community 2 (C2) is predominantly composed of AIDs (7/8), with the exception of recessive mitochondrial ataxia syndrome (AA), while the remaining diseases belonging to Aicardi-Goutières syndrome and its subtypes. This suggests a strong link between recessive mitochondrial ataxia syndrome and Aicardi-Goutières syndrome, offering insight into the positioning of AA diseases within AIIDs. Similarly, Community 6 (C6) is mainly composed of ADs (5/7), with autoimmune lymphoproliferative syndrome and its subtypes dominating, while immunodeficiency 57 - the only AID in this community - consistently clusters with ADs, indicating that, despite its autoinflammatory features, it aligns more closely with ADs. Notably, although each of the 10 communities is dominated by one category, all communities include at least one disease from another category (**Table 3**). This supports recent proposals that AIIDs should not be strictly divided into ADs and AIDs and underscores a broader and more complex disease spectrum. The integration of cross-modal data in our multi-modal AIID network helps enhance our understanding of the underlying mechanisms driving these disease associations.

**Table 3:** Disease counts by categories within robustly associated communities.

Communities	AAs	AIDs	ADs	CAs	Total
C1	2	1	1	0	4
C2	1	7	0	0	8
C3	7	0	6	0	13
C4	3	0	4	0	7
C5	3	1	0	0	4
C6	1	1	5	0	7
C7	6	0	0	0	6
C8	1	1	3	0	5
C9	10	0	0	0	10
C10	0	4	0	1	5

**Figure 3:**
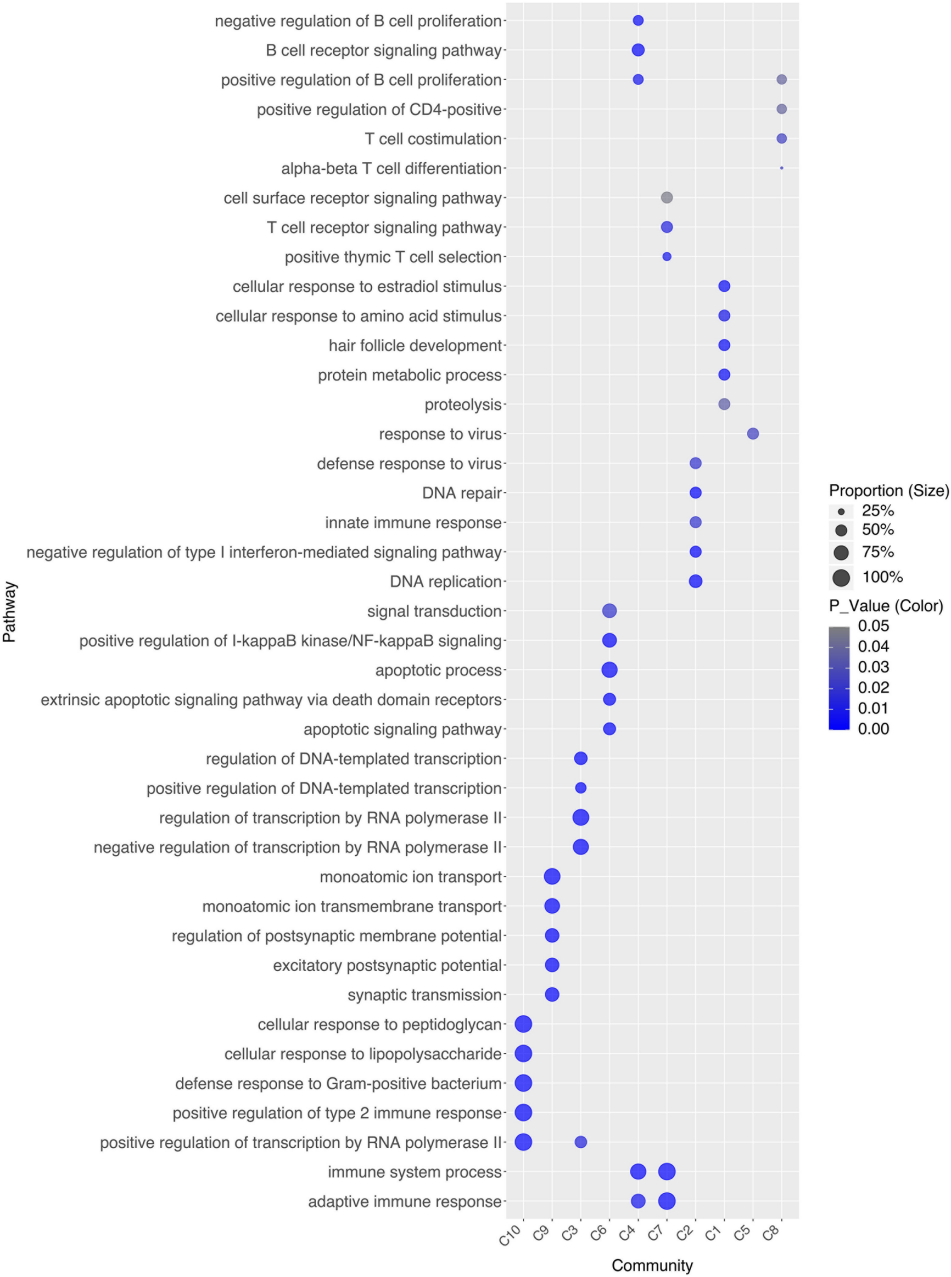
Representative pathways for each disease community.

**Figure 4:**
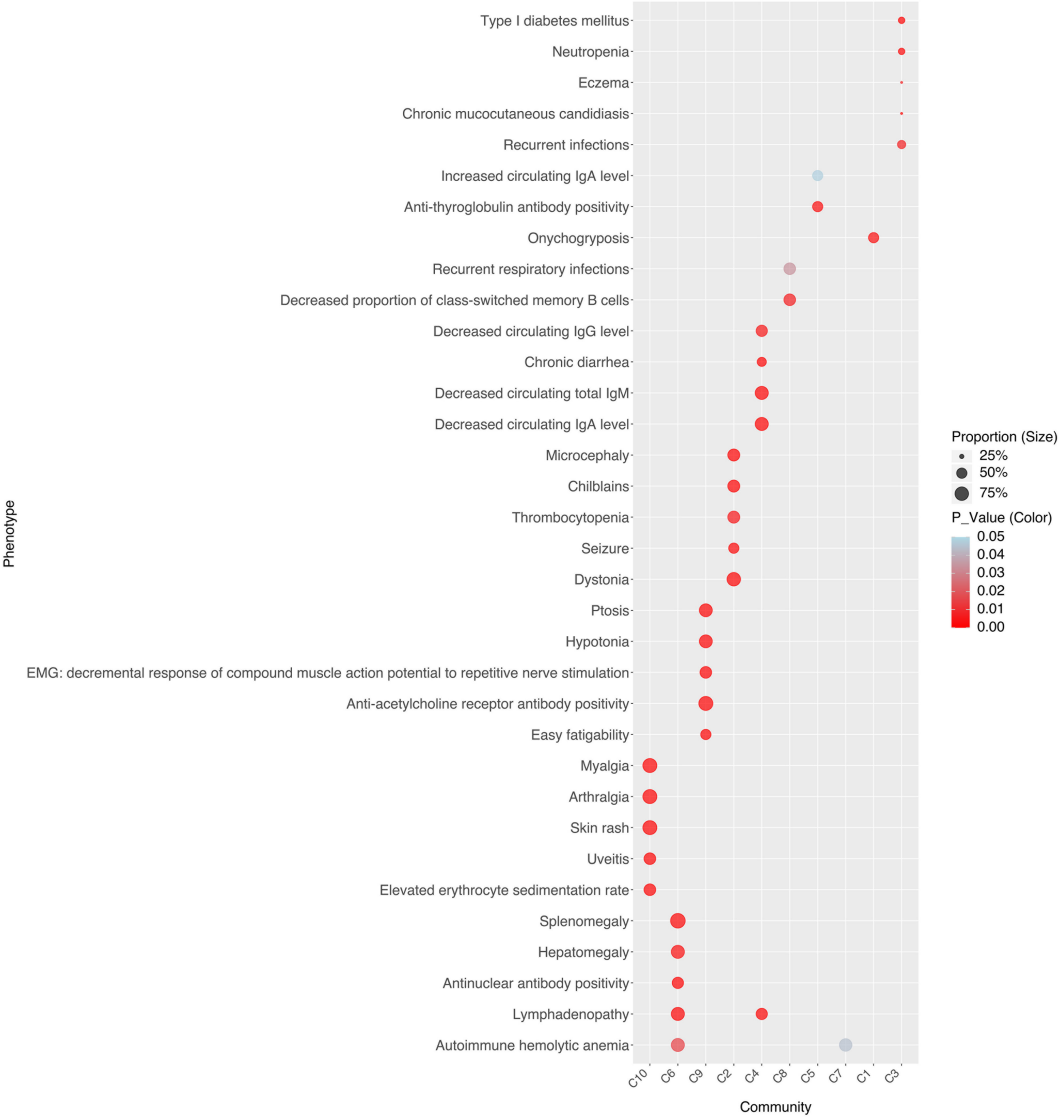
Representative clinical phenotypes for each disease community.

To investigate the drivers of stable associations within disease communities, we identified each community’s representative pathways (**Figure 3**) and clinical phenotypes (**Figure 4**). For instance, C10 comprises four AIDs - familial cold autoinflammatory syndrome 1, cryopyrin-associated periodic syndrome, Muckle-Wells syndrome, and Yao syndrome - and one contested AIID (CA), chronic infantile neurological cutaneous and articular (CINCA) syndrome, suggesting that CINCA syndrome is more closely aligned with AIDs and may merit reclassification. C10 is marked by clinical features such as myalgia, arthralgia, skin rash, uveitis, and elevated erythrocyte sedimentation rate, as well as representative pathways including the positive regulation of type II immune response, cellular response to lipopolysaccharide, transcription regulation by RNA polymerase II, defense response to Gram-positive bacteria, and cellular response to peptidoglycan.

Beyond disease-associated communities, we also paid attention to closely related disease pairs or triplets. Specifically, although most contested AIIDs (CAs) maintain relatively close associations with AIDs, Psoriasis 14, pustular - a contested AIID with an ACS score of 0 - consistently clusters with both Sweet syndrome (AID, ACS = –1) and systemic lupus erythematosus 17 (AD, ACS = 1). This finding, along with evidence of shared classification as nonvasculitic neutrophilic dermatoses and similar responses to granulocyte and monocyte adsorption apheresis, underscores the ambiguity and complexity of AIID classification. Together, these results (**Supplementary Table 4**) highlight the cross-scale interplay of genetic, cellular, and clinical factors underlying AIID comorbidities.

### 3.5 Cross-scale evidence-supported disease association network for core AIIDs

After constructing disease networks for AIIDs at genetic (Gen-DN, 293 diseases), transcriptomic (Trans-DN, 45 diseases), and phenotypic (Phe-DN, 514 diseases) levels, we further derived single-cell-level associations (SC-OADS) by integrating cell type proportions with the Cell Ontology using our ontology-aware disease similarity (OADS) strategy, and built the single-cell-level disease association network (SC-DN). This led to four-layered AIID networks, Gen-DN, Trans-DN, SC-DN and Phe-DN, supported by evidence from the viewpoint of genetic susceptibility, transcriptional dysregulation, immune microenvironment alteration, and clinical manifestation. These four layers collectively cover 10 key AIIDs including Behçet’s disease, Crohn’s disease, juvenile idiopathic arthritis, Psoriasis (PsO), multiple sclerosis, rheumatoid arthritis, SLE, systemic sclerosis (SSc), type 1 diabetes (T1D), and ulcerative colitis (UC). The four layers of disease networks were integrated via the SNF method which combined the input matrices into a single integrated network, capturing both intra- and inter-modality relationships (29) (**Supplementary Figure 3**).

Network reliability was confirmed by comparing disease similarity matrices to a drug-based benchmark, with Trans-DN showing the highest Pearson correlation (r = 0.753), followed by the integrated network (r = 0.707), SC-DN (r = 0.676), Gen-DN (r = 0.582), and Phe-DN (r = 0.550).

**Figure 5:**
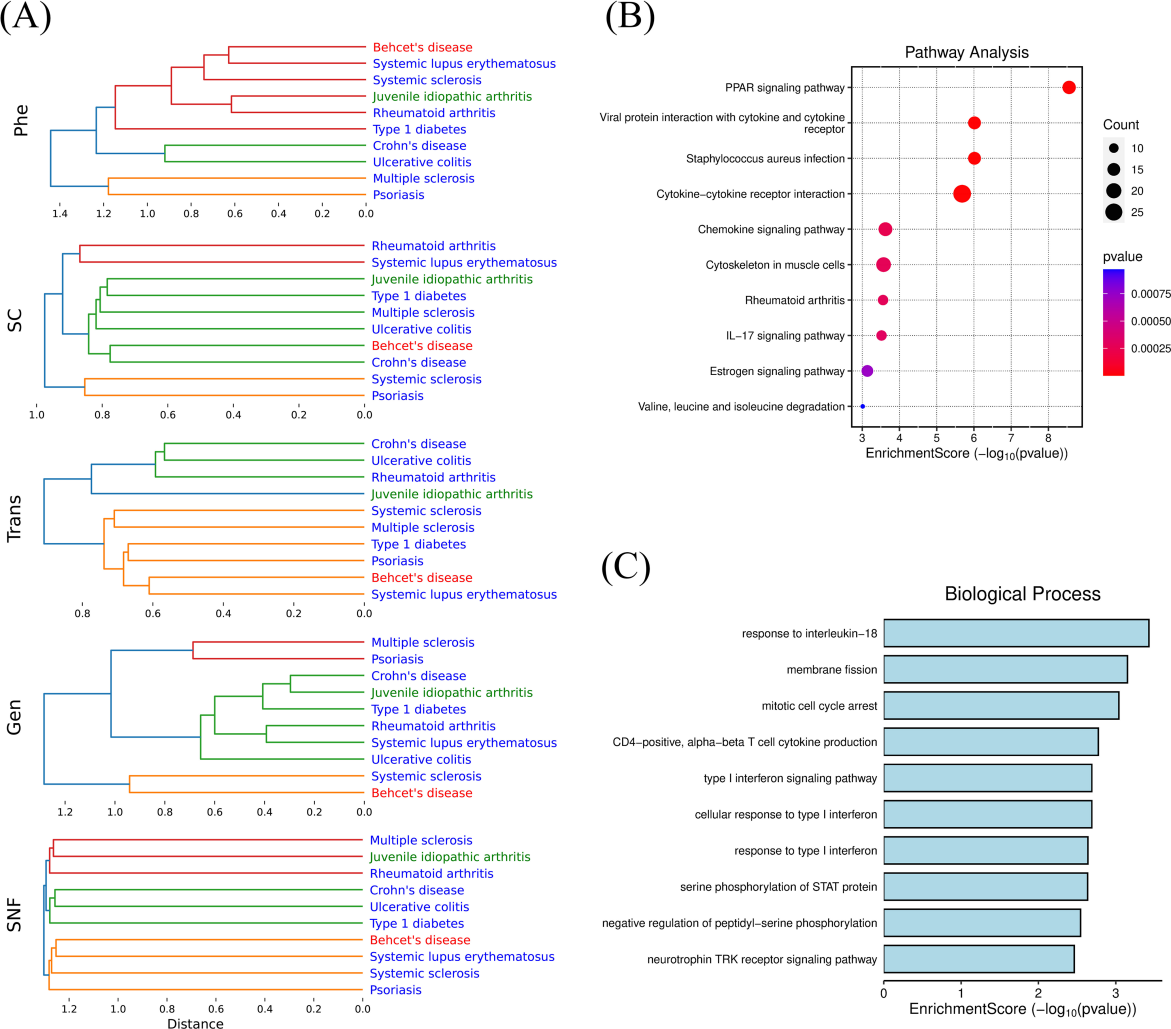
**(A)** Hierarchical Clustering of Single-modal and Integrated Disease Networks; **(B)** Enrichment analysis (KEGG) of overlapping differentially co-expressed genes (DCGs) between systemic sclerosis and psoriasis; **(C)** Enrichment analysis (GO) of overlapping differentially co-expressed genes (DCGs) between Behçet's disease and systemic lupus erythematosus.

Hierarchical clustering revealed unique association patterns among diseases in single-modal networks (**Figure 5A**). For instance, SLE clustered closely with Behçet’s disease in Trans-DN and Phe-DN, but with rheumatoid arthritis in Gen-DN and SC-DN, and Crohn’s disease and UC consistently clustered together, especially in Trans-DN and Phe-DN. In the integrated network, the strong correlations between PsO and SSc, T1D and Crohn’s disease/UC, as well as SLE and Behçet’s disease, provide key clues for exploring shared mechanisms underlying these disease relationships.

To further investigate the mechanisms underlying the association between SSc and PsO, we first applied functional enrichment analysis on genetically associated genes of the two diseases and found that their overlapping pathways involved innate immunity, adaptive immunity, the positive regulation of T cell-mediated cytotoxicity, and so on. Then we traced their differentially co-expressed genes (DCGs) which were used to calculate the transcriptional level disease similarity, and conducted enrichment analyses based on their overlapping genes such as CCL2, CCR7, STAT1, CXCL9, FN1, MMP1, IL1RN, and CD36 (**Figure 5B**). The results revealed significant dysregulation in pathways such as the PPAR signaling pathway, the IL-17 signaling pathway, and chemokine signaling pathway. Ranking the consistency of cell proportions between the two diseases, CD4+ T cells (with a ratio of 1.007) and NK cells (with a ratio of 1.264) were the top two, suggesting their common roles in fibroblast activation and immune cell infiltration in SSc and PsO. At the phenotypic level, both diseases share features such as arthritis and skin involvement.

Previous studies have highlighted the critical role of TGF-β activity in SSc, which impairs PPAR-γ function, contributing to uncontrolled fibroblast activation and progressive fibrosis (38). Meanwhile, PPAR-γ deficiency exacerbates inflammation by disrupting keratinocyte differentiation and amplifying Th17 responses in PsO (39). Notably, PPAR-γ modulates IL-17A production not only in adaptive CD4^+^ Th17 cells (40, 41), but also in innate lymphocytes (e.g., γδ T cells and NK cells) (42, 43). Furthermore, IL-17A itself perpetuates inflammation by inducing NK cell expansion (44). In addition, prior research has established the involvement of IL-17 in both PsO and vascular dysfunction, further supporting its role as another critical mechanism underpinning the association between SSc and PsO (45). Together, these findings delineate a cross-scale pathogenic axis in which PPAR-γ–mediated regulation of both adaptive and innate IL-17 production links genetic susceptibility, cellular dysregulation, and clinical phenotypes in SSc and PsO.

Similarly, we identified differentially co-expressed genes (DCGs) shared by BD and SLE, such as CCR1, GRB10, and IL18R1. Functional enrichment analysis indicated dysregulation of “response to interleukin-18” pathway (**Figure 5C**). IL-18 has been established as a potential therapeutic target for BD (46). Meanwhile, a GWAS study involving 21,758 individuals demonstrated a causal link between IL-18 and SLE risk (47). These findings underscore that IL-18 plays a vital role in both diseases and has the potential to be a common target.

## 4 Discussion

Autoimmune and autoinflammatory diseases (AIIDs) represent a spectrum of disorders driven by diverse immune dysregulation, with significant comorbidity trends reflecting intricate interconnections. However, the underlying mechanisms remain poorly understood. Rapid accumulation of multi-modal data that characterize AIIDs has created new opportunities to measure disease associations and uncover the mechanisms underlying these associations from a cross-scale perspective. In this study, we developed a novel framework for exploring AIID associations by integrating cross-scale, multi-modal data and prior knowledge from continuous biomedical ontologies including GO, Cell Ontology, and HPO.

Curating disease terms from four major disease classification systems (Mondo, DO, MeSH, ICD) and three specialized AIID knowledge bases (the Autoimmune Association, Autoimmune Registry, and the Global Autoimmune Institute), we compiled a comprehensive dataset comprising 484 autoimmune diseases (ADs), 110 autoinflammatory diseases (AIDs), 14 contested diseases (CAs), and 284 diseases associated with AIIDs (AAs). Notably, over half of these ADs and AIDs were recognized as AIIDs by only one knowledge base, highlighting the significant inconsistency in this field and the need to organize and curate the accumulated AIID disease entries.

By designing an ontology-aware disease similarity (OADS) strategy, we constructed multi-layered AIID networks and an integrated network, supported by cross-scale evidence at genetic, transcriptomic, cellular, and phenotypic levels. The disease associations (**Supplementary Figure 1**) and modularity (**Supplementary Figure 2**) of our networks display significant complementarity across multi-layered disease networks, supporting the necessity of multi-modal study of disease association for AIIDs. In recent years, the rapid growth of multi-omics data and standardized clinical phenotypic information has greatly advanced cross-scale studies spanning “molecule-cell-tissue-organ” levels (13, 48). Until now, significant efforts have been devoted to addressing the challenges of high dimensionality, noise, and sparsity in multi-modal data (49, 50); in contrast, the effective incorporation of prior knowledge has long been overlooked. Although certain ontologies, such as GO, have been widely used in biomedical studies, the potential of continuous and unified ontologies has not been fully realized (51). Our OADS methodology provides a reasonable and feasible demonstration of integrating multi-modal data with biomedical rules, generating mechanistically interpretable results that encompass genetic susceptibilities, transcriptional dysregulation, alteration in immune microenvironment, and clinical phenotypes. Notably, this is the first time that single-cell data has been integrated into a disease association study among the four layers. By incorporating cell composition information, we enhance the characterization of the cellular microenvironment, bridging the gap between the molecular characterization of diseases and clinical phenotypes. This integration is crucial for understanding the cross-scale information flow during disease pathogenesis.

**Figure 6:**
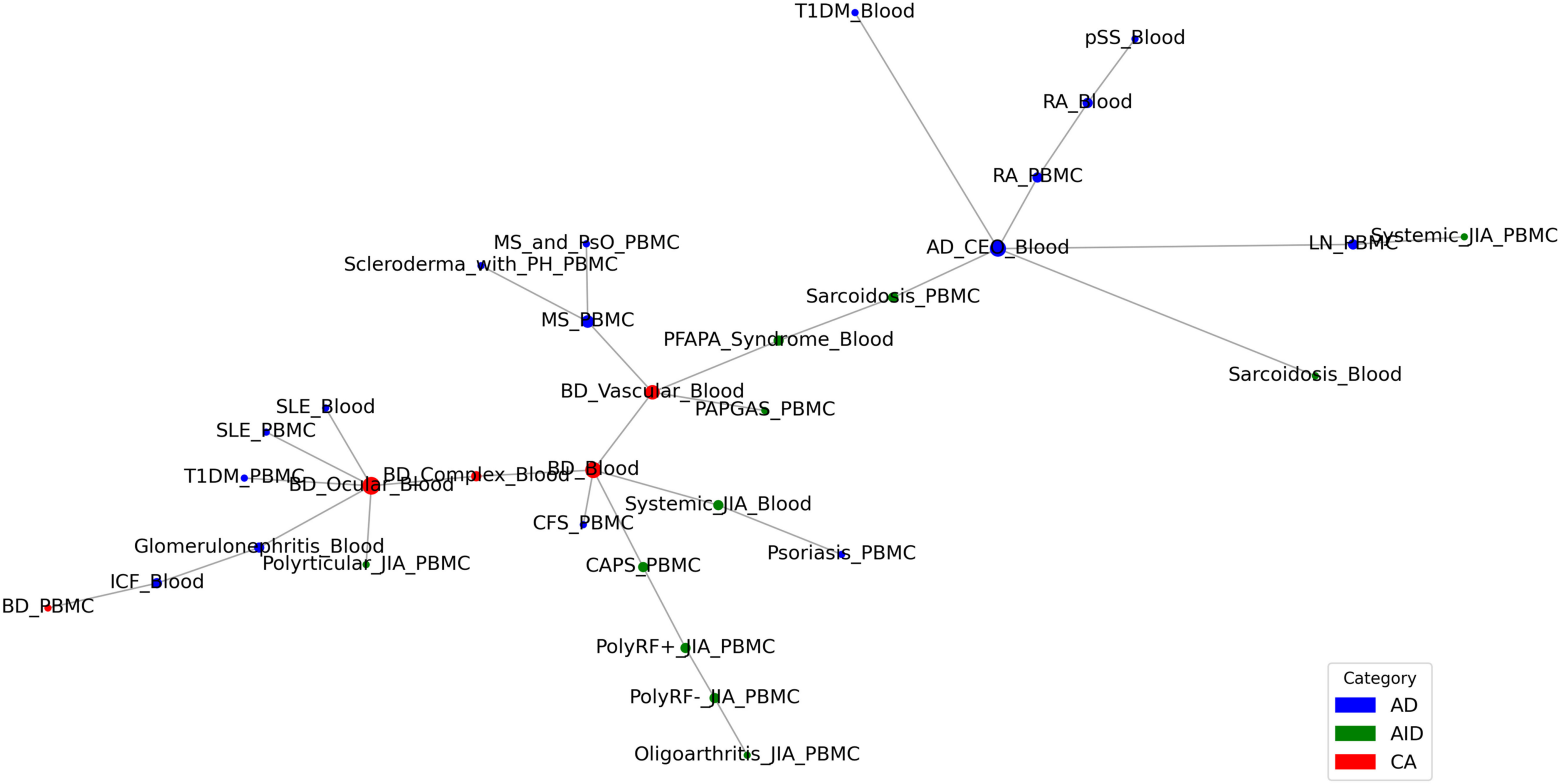
Disease Trajectory Inference Based on Trans-DN.

Emerging evidence suggests that AIIDs exist on a continuum rather than as discrete entities: ADs and AIDs. “Mixed-pattern” AIIDs especially challenge the traditional binary classification and necessitate a more nuanced understanding of AIID pathogenesis (1, 5). In order to explore the clustering modes and evolutionary patterns among associated AIIDs, we performed disease trajectory inference using MST-based analyses on a transcriptomic network (Trans-DN) involing 45 diseases (**Figure 2**). As shown in **Figure 6**, the trajectory exhibited multi-branching, clustering within ADs (indicated by the circle around the blue nodes) or within AIDs (indicated by the circle around the green nodes), as well as AD-AID or AID-AD transitions (AD-AID neighbors) as well. This pattern can also be observed in the phenotypic (Phe-DN), genetic (Gen-DN) networks (**Supplementary Figures 4A, B**). These results suggest that the relationship patterns among AIIDs are much more intricate than a binary or linear spectrum framework, such as “AD-mixed-AID”. Taking Behçet’s disease (BD) – classified as Contested (CA, **Figure 1A**) - as an example, BD displayed strong associations with systemic lupus erythematosus (SLE, a classic AD) within our integrated network (**Figure 5A**).Trajectory analysis in Trans-DN suggested that BD is closely associated with both ADs and AIDs but exhibits a greater similarity to ADs. Likewise, systemic-onset juvenile idiopathic arthritis (So-JIA), although traditionally categorized as an AID, exhibited significant associations with PsO (AD) and BD. These findings align with the dynamic interplay between adaptive and innate immune mechanisms in AIID pathogenesis.

This study integrates multi-modal data and continuous biomedical ontologies to deepen the understanding of AIID pathogenesis within the context of disease communities. It offers new insights for diagnosis and management while demonstrating potential for translational applications such as drug repurposing and therapeutic target discovery. However, current methods are limited by the incomplete coverage of public datasets and the heterogeneity of multi-source data (differences in resolution, dimensions, and weight distribution). Future efforts should also focus on developing and customizing multi-modal data integration methods that combine biomedicine rules with deep learning, and validating findings through large-scale clinical studies. This technological framework holds broad applicability for diseasome research on complex diseases.

## Data Availability

The original contributions presented in the study are included in the article/**Supplementary Material**. Further inquiries can be directed to the corresponding author.

## Supplementary material

Supplementary Figure 1
**(A)** Degree Distribution Fitting with Poisson of Phe-DN; **(B)** Degree Distribution Fitting with Poisson of Gen-DN; **(C)** Degree Distribution Fitting with Poisson of Transn-DN; **(E)** Disease pair ranking transitions across different modalities; **(F)** Disease Category Composition within Clusters (Clusters dominated by a single category are color-coded to match the respective category).

Supplementary Figure 2 Evaluation of Clustering Consistency **(A)** Sankey diagram of clustering results across different modalities and methods; **(B)** Heatmap of Adjusted Rand Index (ARI) values for clustering consistency across modalities and methods.

Supplementary Figure 3 Integrated disease network (SNF-DN).

Supplementary Figure 4
**(A)** Disease Trajectory Inference Based on Phenotypic Disease Network (Phe-DN); **(B)** Disease Trajectory Inference Based on Genetic Disease Network (Gen-DN).

Supplementary Table 1 Repository of AIIDs, Modal Data, and ACS Scores.xlsx. This table provides detailed information on autoimmune and autoinflammatory diseases (AIIDs), including multi-modal data sources and AIID Classification Score.

Supplementary Table 2 Similarity Matrices Across Modalities.xlsx. A comprehensive presentation of similarity matrices derived from different modalities, illustrating cross-modal disease relationships.

Supplementary Table 3 Clustering Results and Network Characteristics.xlsx. Summarizes clustering outcomes, network attributes, and topological features identified across multi-modal disease association networks.

Supplementary Table 4 Stable Associations Across Modalities and Clustering Methods.xlsx. Highlights robust disease associations identified consistently across various data modalities and clustering techniques, offering insights into shared patterns and cross-modal stability.
