# Supplementary figures and images for: Disease association study of Autoimmune and autoinflammatory diseases by integrating multi-modal data and hierarchical ontologies
Source: Front Immunol. 2025 Jun 4;16:1575490. doi: 10.3389/fimmu.2025.1575490 (PMC12174166; doi:10.3389/fimmu.2025.1575490)

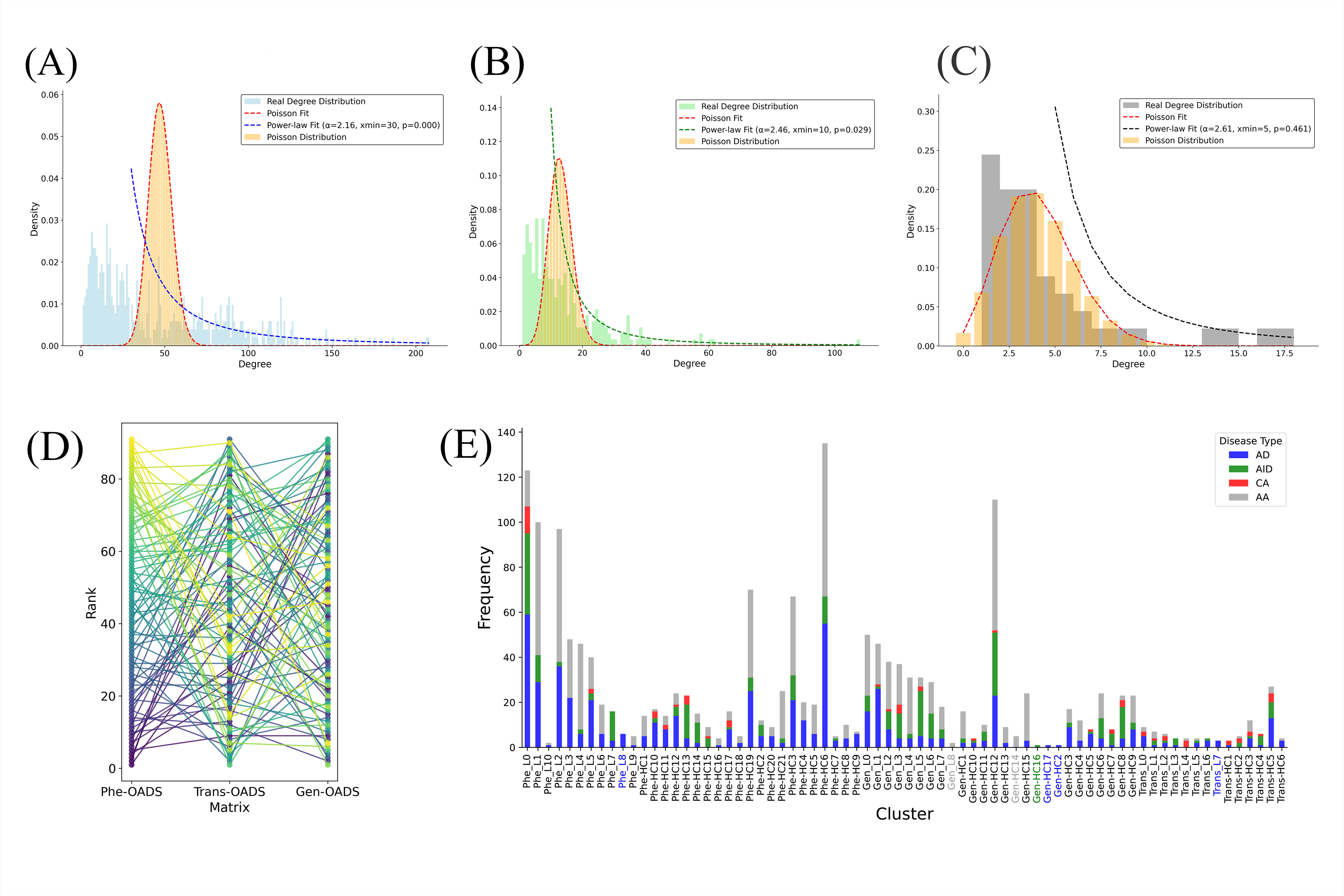

Supplement: Supplementary Figure 1 — (A) Degree Distribution Fitting with Poisson of Phe-DN; (B) Degree Distribution Fitting with Poisson of Gen-DN; (C) Degree Distribution Fitting with Poisson of Transn-DN; (E) Disease pair ranking transitions across different modalities; (F) Disease Category Composition within Clusters (Clusters dominated by a single category are color-coded to match the respective category). [file Image1.tif]

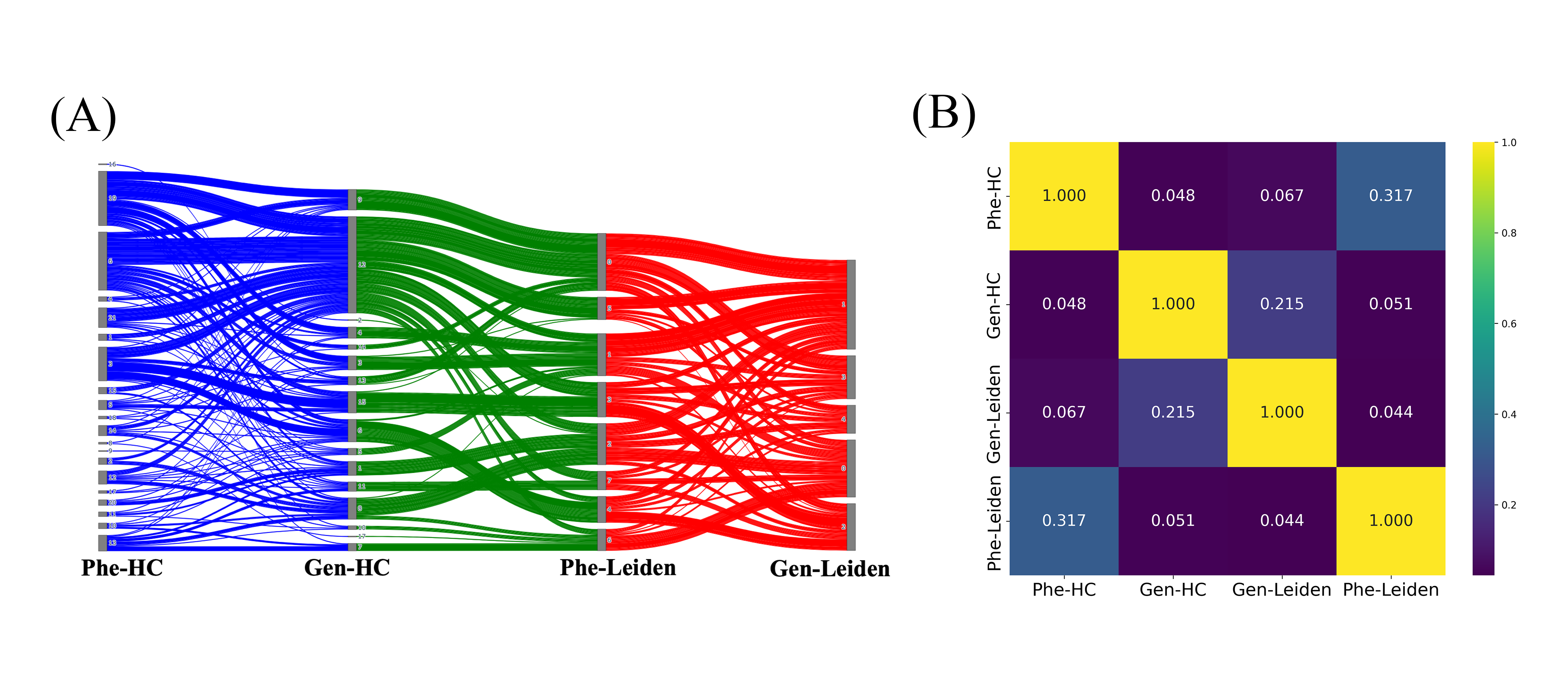

Supplement: Supplementary Figure 2 — Evaluation of Clustering Consistency (A) Sankey diagram of clustering results across different modalities and methods; (B) Heatmap of Adjusted Rand Index (ARI) values for clustering consistency across modalities and methods. [file Image2.tif]

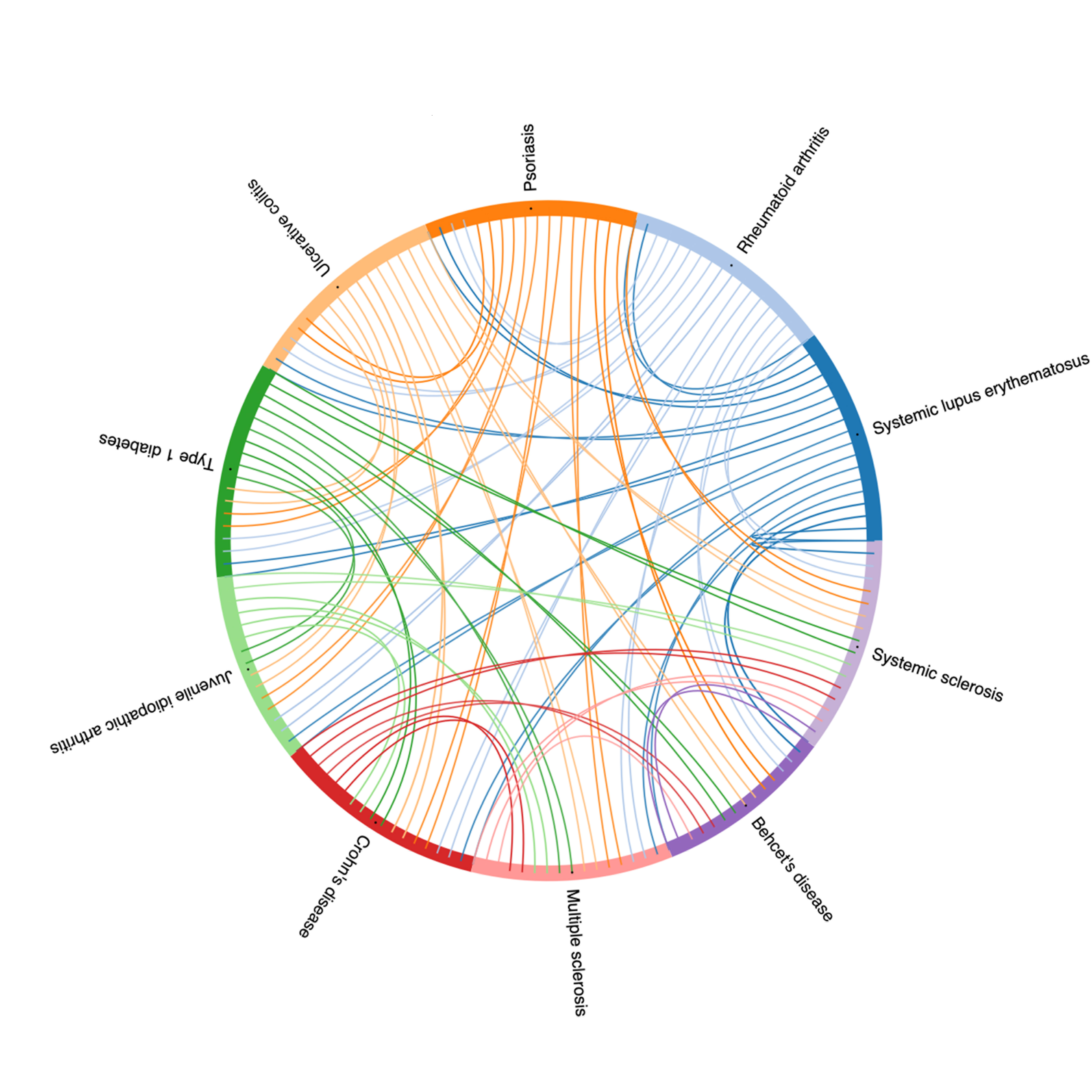

Supplement: Supplementary Figure 3 — Integrated disease network (SNF-DN). [file Image3.tif]
